# An Overview of the Latest Progress in Internal Surface Finishing of the Additively Manufactured Metallic Components

**DOI:** 10.3390/ma16103867

**Published:** 2023-05-21

**Authors:** Farideh Davoodi, Mohammad Taghian, Giuseppe Carbone, Abdollah Saboori, Luca Iuliano

**Affiliations:** 1 Department of Materials Engineering, Isfahan University of Technology, Isfahan 84156-83111, Iran; farideh.davoodi@poliba.it; 2 Department of Mechanics, Mathematics and Management, Politecnico di Bari, V.le Japigia, 182, 70126 Bari, Italy; giuseppe.carbone@poliba.it; 3 Integerated Additive Manufacturing Center (IAM), Department of Management and Production Engineering, Politecnico di Torino, Corso Duca degli Abruzzi 24, 10129 Torino, Italy; mohammad.taghian@polito.it (M.T.); luca.iuliano@polito.it (L.I.)

**Keywords:** additively manufacturing, roughness analysis, internal surface finishing, magnetic abrasive finishing, abrasive flow machining, fluidized bed machining, cavitation abrasive finishing, electrochemical machining

## Abstract

Fast progress in near-net-shape production of parts has attracted vast interest in internal surface finishing. Interest in designing a modern finishing machine to cover the different shapes of workpieces with different materials has risen recently, and the current state of technology cannot satisfy the high requirements for finishing internal channels in metal-additive-manufactured parts. Therefore, in this work, an effort has been made to close the current gaps. This literature review aims to trace the development of different non-traditional internal surface finishing methods. For this reason, attention is focused on the working principles, capabilities, and limitations of the most applicable processes, such as internal magnetic abrasive finishing, abrasive flow machining, fluidized bed machining, cavitation abrasive finishing, and electrochemical machining. Thereafter, a comparison is presented based on which models were surveyed in detail, with particular attention to their specifications and methods. The assessment is measured by seven key features, with two selected methods deciding their value for a proper hybrid machine.

## 1. Introduction

After presenting a new method of fabrication for three-dimensional plastic models by Kodama in 1981, several different layered manufacturing techniques have been developed [1,2]. After that, different kinds of materials, such as polymers, ceramics, and metal powders, have been employed to produce a range of functional end-products [2,3]. After these developments, the cooling channels have been designed in a complex form by using a computer-aided design (CAD) model [4], while traditionally these cooling channels have been formed by straight-drilled holes [5]. 

According to ISO/ASTM 52900:2015 (E), additive manufacturing (AM) is a process of joining materials to make parts from 3D model data, usually layer upon layer, instead of subtractive manufacturing and formative methodologies [6]. In the case of direct AM processes, such as powder bed fusion (PBF) [7,8] and directed energy deposition (DED) processes [9,10], the final shape and properties of a part are achieved in a single step through a melting/solidification of similar materials without any post-processing necessity [11,12]. In indirect AM processes, such as binder jetting (BJT), the components are only shaped by AM processes. Further post-processing, such as debinding and sintering, is required to consolidate the material and obtain the final density, geometry, and properties. According to the final requirements, some post-processing such as thermal treatment, surface finishing, and machining in the direct and indirect AM processes will be implemented on the as-built parts [13,14]. However, it has been revealed that the internal surface of the cooling channels after fabrication by AM techniques has a low quality due to the presence of partially/unmelted particles, adhered powder, and pores [15,16]. The internal channels with poor surface quality could induce undesired boundary layer turbulence in the gas or fluid flowing inside them. In some particular components, such as turbine spray nozzles, waveguides, and hydraulic manifolds, the quality of the internal surface finish plays a key role.

Nonetheless, even the internal surface finishing is difficult due to the limitations of reaching the internal parts and holes of the products. Conventional machining methods like grinding, honing, and lapping are unsuitable for internal surface finishing because they are time-consuming, labor-intensive, low quality, and have geometrical limitations [17]. Thus, it is easy to conclude that conventional techniques can never achieve, from an engineering point of view, a perfectly smooth surface for the internal channels. Therefore, with AM technology’s progress in producing complex shape components consisting of internal channels, the level of interest in advanced surface finishing increases.

**Figure 1:**
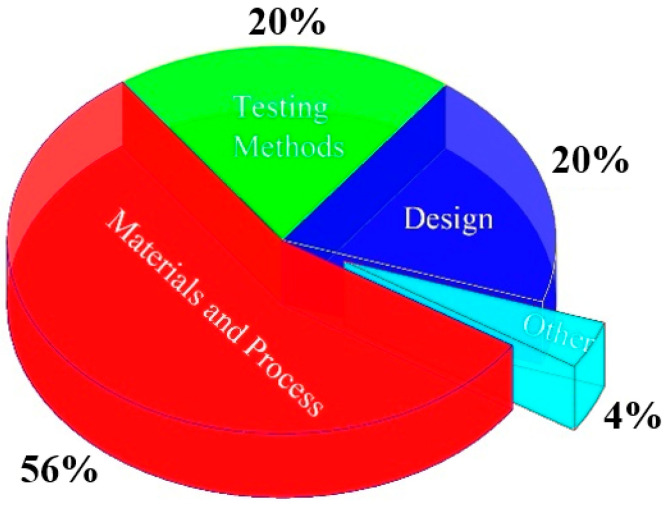
Breakdown of the active ISO/ASTM standard on AM.

ASTM Committee F42 on Additive Manufacturing Technologies has been active since 2009 and is composed of subcommittees addressing specific segments such as materials and processes, design, test methods, and so on. The purpose of the committee is the promotion of knowledge, stimulation of research, and implementation of technology through the development of standards for additive manufacturing technologies. These standards are expected to play a significant role in all aspects of additive manufacturing technologies. As shown in Figure 1, which is based on the current active standards, most of the standards are from the materials and processes, as can be expected. Instead, only less than 4% of these standards are about the other parts, such as surface finishing of AM metals. Therefore, this graph clearly shows the lack of deep research and the novelty of the field of surface finishing on metal AM parts, and several research and development opportunities can be explored [18].

The roughness of a surface is affected by various process parameters, such as the energy source, scan speed, scan pattern, hatch spacing, build orientation, and channel diameter. The side surface roughness is less affected by process factors than the top surface roughness, and optimization rarely reduces the side surface roughness. Even with the best process factors, AM components have linear surface roughnesses in the 5 to 15 μm range [19,20]. It is highly desirable for medical, aeronautical, and biosensor components to have a surface roughness (*Ra*) of less than one micron before they are used in practical applications [21,22]. Using mechanical energy-based finishing processes, surface roughness can be effectively reduced using mass finishing, sand blasting, shape adaptive grinding, etc. [23]. However, these finishing processes create complex surface textures and lay directions, making it challenging to finish complex features such as lattice systems and other complex structures. On the other hand, thermal energy-based finishing processes leave recast and oxide layers on the polished surface and produce residual tensile stress due to rapid heating and solidification. For example, Temmler et al. [24] employed laser polishing based on thermal energy to finish the surface of the tool steel materials. In this case, a high residual tensile stress of up to 926 MPa was found after this polishing process. The advantage of non-traditional finishing processes is that the surface roughness patterns and residual stresses are rarely produced on the polished surface. Additionally, these processes produce consistent, predictable, and reproducible results [25].

Nowadays, the need for precise internal surface finishing attracts enormous interest from researchers to contribute to developing innovative and hybrid methods for complex shapes [17,26]. For instance, Nagalingam et al. [27] analyzed the surface quality of internal channels in IN 625 parts before and after using a multi-jet hydrodynamic approach. They found that using the multi-jet hydrodynamic approach can improve the surface quality of IN 625 by up to 90%. In another work, Nagalingam et al. [28] studied the effect of hydrodynamic cavitation abrasive finishing on the surface finish quality of additively manufactured channels. They reported that internal surface finishing using cavitation-aided microparticle abrasion results in a 90% improvement in the surface finish quality of internal channels. Guo et al. [29] developed a novel rotating-vibrating magnetic abrasive polishing method to finish a double-layered internal channel of IN 718 produced by the laser powder bed fusion (L-PBF) process. Han et al. [30] evaluated the influence of the abrasive flow machining (AFM) method on the surface finish of the L-PBF-produced conformal channels.

The objective of this paper titled is to provide a comprehensive review of the current state of research and development in the field of internal surface finishing of metallic components produced using additive manufacturing (AM) techniques. This paper aims to highlight the recent progress and advances in various methods and techniques used for the internal surface finishing of AM metallic components. Far too little attention has been paid to comparing the available methods and prioritizing them according to their efficiency, cost, part size limitation, and material limitation. Hence, in this work, the authors address the practical results of different surface finishing methods, such as magnetic abrasive finishing, abrasive flow machining, fluidized bed machining, cavitation abrasive finishing, and electrochemical machining, considering the recent outcomes. Regarding the methodology, the scope of this paper, compared to previous reviews, is brought up with more comprehensive frontier models since the authors aim predict which models could combine in the right way. To this aim, first, a specific domain of models in the literature has been reviewed. These features are directly extracted from the literature and represent common surface finishing challenges. Finally, a score-based comparison is presented as a novel work to rank the priorities and define the combined system. In addition, this paper identifies the challenges and opportunities for further research and development.

### Research Methodology

**Figure 2:**
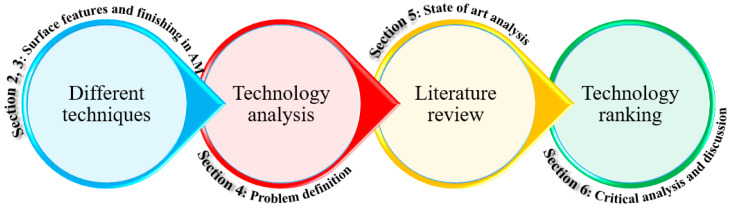
Methodology of the literature survey.

A multi-layer chain in Figure 2 represents the methodological approach and the structure of the paper’s content. The papers have been filtered regarding their basic assumptions, objectives, strengths and limitations, essential elements, and applications based on a four-step procedure:

(I) Problem definition through state-of-the-art analysis of the achieved surface characteristics of the as-built parts through the metal AM processes and related internal surface finishing techniques;
(II) Considering the table of contents of several significant journals from 2005 to early 2023;
(III) Verifying the reference list of highly cited papers based on keywords;
(IV) Surveying the publications of outstanding scholars within surface finishing methods to find the most influential papers in this area.

These four steps have given a superset of more than 100 papers, further filtered for relevance to internal surface finishing. Building on this paper repository, a literature review is implemented in the third echelon, along which the articles are investigated in detail through the five key issues. The aim is to recognize a set of criteria that can confirm the possibility of process combination for obtaining the best possible surface roughness in complex-shaped components with different materials.

## 2. Surface Features in PBF Parts

In the PBF process, the components must be oriented between ±90° with respect to the building plate, forming surfaces with different qualities. It is well documented that poor surface roughness and inaccurate internal channels are two inherent features of additively manufactured components [31,32,33]. Table 1 compares the as-built surface roughness of metallic parts produced using different PBF machines.

As shown in Table 1, the surface roughness of metallic parts produced via direct AM depends on the material and machine and lies in the range of 8–80 μm. This wide range can increase the uncertainty of the mechanical properties of the parts. However, it should be underlined that surface roughness, particularly of internal surfaces, plays a key role when placed in lubrication, wear, and friction applications [38,39,40]. Moreover, it is reported that surface quality could strongly affect the fatigue life of the metal AM parts. In fact, by increasing the surface roughness, the change of crack initiation increases, reducing the components’ fatigue lives. Hence, it can be expected that AM parts, compared to conventionally manufactured ones, have lower fatigue life. Li et al. [41] evaluated this aspect by comparing the fatigue life of PBF and cast parts. Interestingly, they found that the fatigue life of the AM parts was much higher than those conventionally machined. This finding proved that the fatigue life of each component could be severely influenced by surface roughness and internal defects. Hassanin et al. [42] studied the correlation between the build orientation and the surface quality of the additively manufactured Ti-6Al-4V components of internal channels. They found that building at 0° results in almost 50% lower dimensional accuracy due to the formation of surface roughness equal to 10–80% of the diameter of internal channels. Snyder et al. [43] studied this aspect in more detail using an X-ray-computed tomography system. Their outcomes demonstrated that the source of this low dimensional accuracy in the internal channels is the formation of ball-like asperities at the down skin of the internal channels. This poor surface quality in the AM parts, particularly in the down skin surfaces, is because of the loosely adhered powder particles or defects formed during the process. The lowest surface roughness that can be achieved via PBF processes is the one that comes out of the laser-based PBF process, which is 5–10 μm. Therefore, the typically required surface roughness for most applications, which is less than 1 μm, cannot be achieved through the PBF methods. All in all, several factors can affect the final surface roughness of an AM part; (I) loosely adhered particles [44], (II) partially melted particles [45,46], (III) surface pores [47], (VI) stair casing [48,49], and (V) balling melts [50]. Despite several efforts that have been made to eliminate these defects through process parameter optimization, still, some post-surface finishing is mandatory in most cases before placing them in practical applications [51].

## 3. Internal Surface Finishing

Conventional finishing processes refer to the traditional methods of surface finishing that have been used for many years in manufacturing. These processes are typically mechanical, such as grinding, sanding, and polishing. Grinding is a process in which an abrasive material, such as a grinding wheel or belt, is used to remove material from the surface of a part or component. This process is often used to achieve a smooth, uniform surface finish or to remove any unwanted material or defects from the surface [52]. Sanding process involves the use of abrasive materials, such as sandpaper or abrasive pads, to remove material from the surface of a part or component. Sanding is often used to achieve a smooth, uniform surface finish or remove any scratches, blemishes, or other imperfections [53]. Another technique is polishing, which involves using a polishing compound and a buffing wheel to create a smooth, reflective surface finish. This process is often used to improve the aesthetics of a part or component or to achieve a specific surface finish requirement [54]. Chemical-based finishing processes, such as etching, may also be considered conventional finishing processes, which involve the use of chemical reactions to modify the surface of the part or component, often for decorative or corrosion-resistant purposes [55]. While conventional finishing processes have been widely used and well established, they may not always be suitable for finishing complex features or achieving precise surface finishes. In recent years, non-conventional finishing processes have emerged as promising alternatives to conventional methods, particularly for parts and components produced using additive manufacturing techniques.

Non-conventional surface finishing is a crucial post-processing step in producing complex shape components with internal channels. The internal surface roughness can affect the dimensional accuracy of the channels and flow characteristics during flow transportation. To date, several studies have proposed different types of surface finishing that can be applied in finishing the internal surfaces of the channels and tubes. For instance, the magnetic abrasive finishing (MAF) method has been introduced by Shinmura et al. to finish the internal surface of stainless tubes [56]. Kim et al. [57] developed a magnetic abrasive jet machining system to finish the inner surfaces using a magnetic abrasive and a fluid mixture. However, every method has limitations in the surface finishing of some specific internal channels, such as blind holes and internal channels with protuberances.

**Figure 3:**
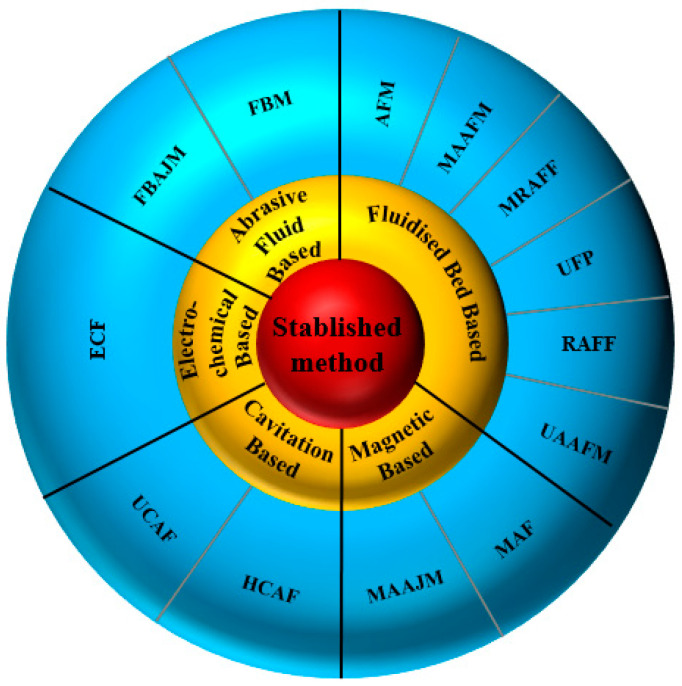
Categories of different internal surface finishing methods. (AFM: abrasive flow machining; MAAFM: magnetic-assisted abrasive flow machining; MRAFF: magnetorheological abrasive flow finishing; UFP: ultrasonic flow polishing; RAFF: rotational abrasive flow finishing; UAAFM: ultrasonic-assisted abrasive flow machining; MAF: magnetic abrasive finishing; MAAJM: magnetic-assisted abrasive jet machining; HCAF: hydrodynamic cavitation abrasive finishing; UCAF: ultrasonic cavitation abrasive finishing; ECF: electrochemical finishing; FBM: fluidized bed machining; FBAJM: fluidized bed abrasive jet machining.).

On the other hand, the necessity of finishing different kinds of internal channels has forced researchers to find other solutions. According to the literature, the most well-known and feasible methods that can be used to finish the surface of internal passages in the complex-shaped components are abrasive flow-based, magnetic abrasive-based, fluidized-based, cavitation-based, and electrochemical-based finishing (Figure 3). Thus, these applicable internal surface finishing methods are reviewed in this article, and all the developments in those methods are reported.

### 3.1. Abrasive Flow Finishing (AFF)

**Figure 4:**
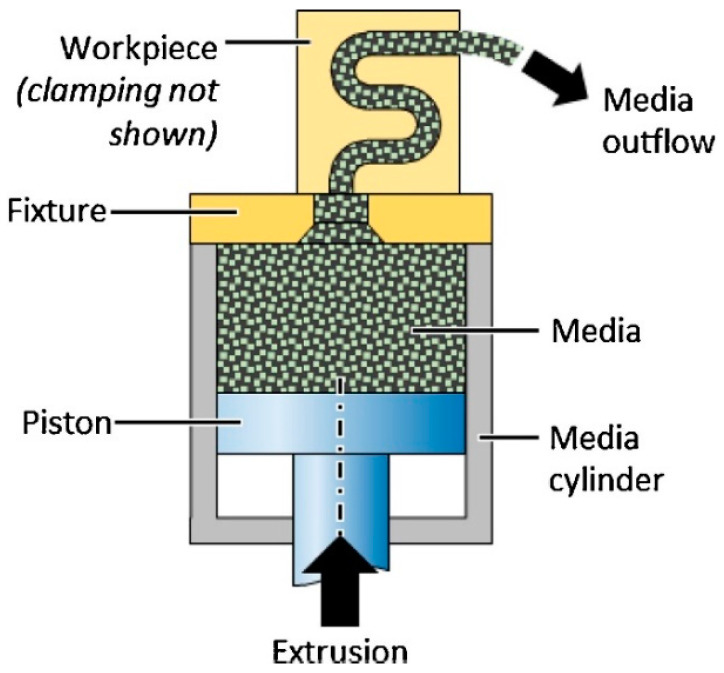
Schematic of abrasive flow machining process [59].

AFF or AFM, developed explicitly for internal surface finishing, is a new method to achieve high precision on the internal surface and can reach a roughness of 0.2 μm or less [26]. In the beginning, this method was used by Kim for the sake of internal deburring [57]. After that, Yin et al. [58] used this method to polish the microchannels of mechanical components. In principle, AFM is an advanced finishing technique to finish the internal surfaces by flowing a semi-solid, visco-elastic, abrasive-laden medium under pressure up to 220 bar into the channels (Figure 4).

**Figure 5:**
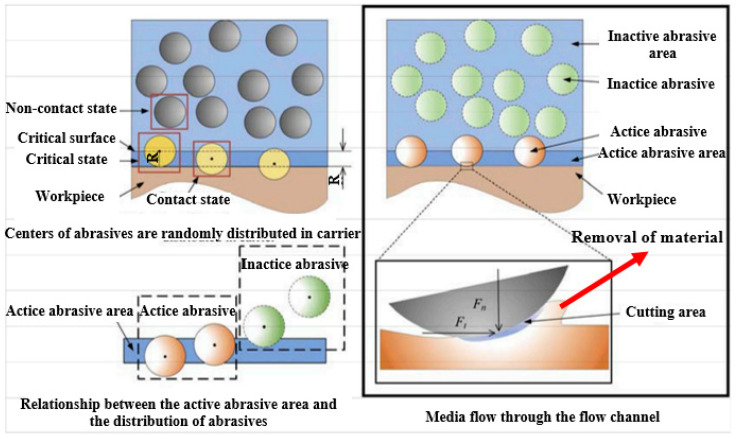
Schematic of the material removal in AFM technique [61].

In this technique, silicon carbide or alumina usually is used as the abrasive material, whereas polyborosilixane is employed as the noncorrosive medium to finish the surface. Micro-cutting and micro-ploughing are two main material removal mechanisms in the AFM method that results in a surface finish in the range of micrometers to nanometers [60]. The flow of the highly viscous media forms a radial force against the surfaces of the internal channel. Then, a fraction of abrasive agents starts to penetrate the surface of the channel and remove a layer of 1–10 μm to reduce the internal surface roughness (Figure 5).

**Figure 6:**
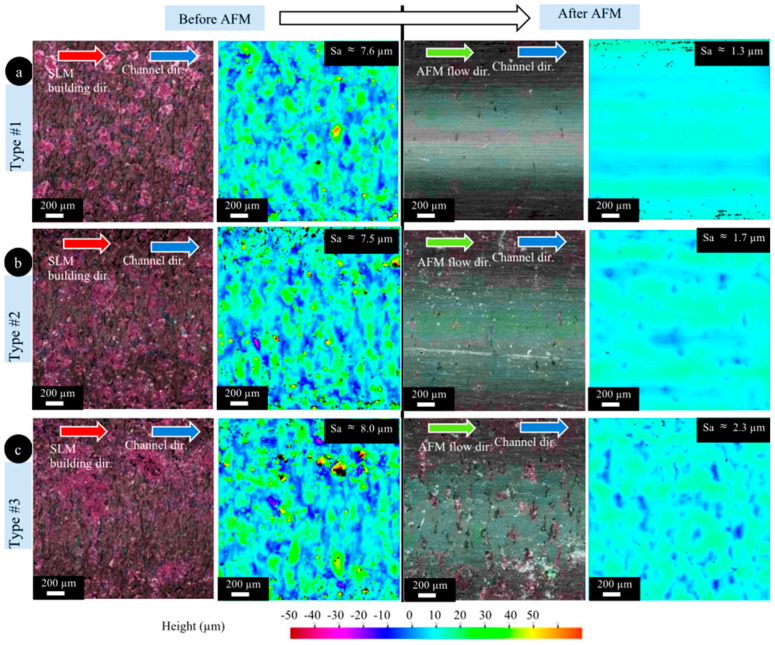
Optical images and height color maps of straight conformal channels in the as-built state and after the AFM process; ((**a**–**c**) refer to different types of conformal cooling) [30].

The material removal rate depends on several factors: media flow rate, viscosity, abrasive particle size, abrasive concentration, particle density, particle hardness, and workpiece hardness. Nevertheless, AFM can be applied to various metals such as titanium, superalloys, and hardened and difficult-to-machine materials [26,60]. For instance, Han et al. [30] used the AFM technique to finish the conformal cooling channels made of maraging steel produced via the L-PBF process. In their work, different conformal cooling channels, including strength/helical channels, were produced by the L-PBF process and then finished by the AFM process. Their outcomes proved that AFM effectively improves the surface quality of all conformal cooling channels produced via the L-PBF process. The results of the straight channels in the as-built state and after finishing the operation are presented in Figure 6. As can be seen in Figure 6, the surface roughness of the straight conformal channel after AFM is reduced from 7–8 μm in the as-built state to 1–2 μm. Moreover, the L-PBF surface texture is removed after the finishing operation.

Recently, Hashmi et al. [62] examined the effects of AFM process parameters on improving the surface roughness of the FDM-printed hollow truncated cone shape. The results of the Taguchi design showed that the media viscosity and finishing time are the most effective process parameters. It was found that the maximum improvement occurred at a media viscosity of 2.10 Pa.s, a finishing time of 30 min, and a layer thickness of 0.2 mm, resulting in a 94.26% improvement in the surface roughness (from 20.93 μm to 1.20 μm) of the workpiece.

**Figure 7:**
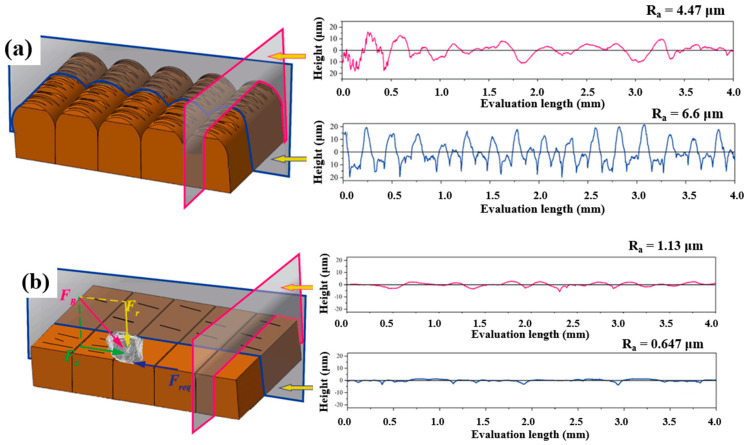
Schematic diagram of the additively manufactured copper and related surface roughness profiles (**a**) before and (**b**) after surface finishing in a medium consists galactomannan polymer, glycerol solution, and abrasive particles [63].

In another work by Basha et al. [63], an alternative long-lasting and noncorroding abrasive medium consisting of galactomannan polymer, glycerol solution, cross-linker, and abrasive particles was indigenously developed for the finish atomic diffusion additively manufactured pure copper. Increasing extrusion pressure and the cycle numbers resulted in a more significant amount of material removal, while increasing the abrasive mesh size led to a smaller amount of material removal. With increased cycle number and extrusion pressure, surface roughness improved within the longitudinal direction, but the roughness increased with the larger size of the abrasive mesh. A schematic illustration of atomic diffusion additively manufactured copper before and after surface finishing in the mentioned medium is presented in Figure 7. An opposite effect of the particle size was observed in the work by Petare et al. [64]. They showed that increasing abrasive particle size can decrease the surface roughness of spur and straight bevel gears.

**Figure 8:**
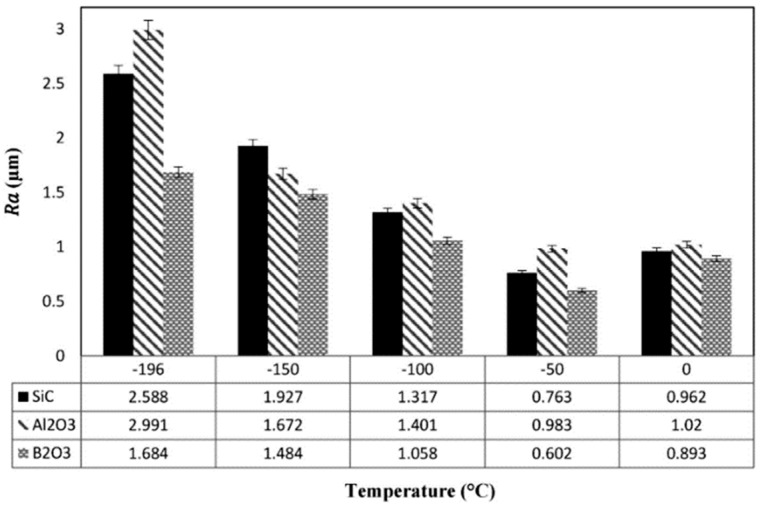
The surface roughness values at different temperatures with three abrasive particles [65].

Seyedi et al. [65] employed the AFM technique to study the effect of Al_2_O_3_, SiC, and B_2_O_3_ particles and different temperature ranges on maraging steel’s roughness and wear properties. The findings showed that the media containing Al_2_O_3_ particles led to a surface with higher roughness than other media. In addition, the variation of surface roughness at different temperatures can be observed in Figure 8.

However, it is reported that very high pressures of abrasive flow (>220 bar) inside the channels damage the surface of the thin-walled channels. Therefore, it is necessary to control the pressure of the abrasive flow inside the channels and control the cutting forces during the finish operation. For this reason, magnetic-based AFM methods have been developed and introduced. Magnetic-assisted AFM (MAAFM) and magnetorheological abrasive flow finishing (MRAFF) are examples of these magnetic-based AFM methods [66,67]. In fact, in these methods, magnetic forces control the cutting forces and material removal by controlling the working fluid. MAAFM aims to increase the number of active abrasive grains and provide extra finishing energy to increase the finishing efficiency.

In general, in MAAFM, applying a magnetic field makes it possible to pull the abrasive media that includes ferromagnetic abrasive particles to the sides, consequently enhancing the number of active abrasive grains. Instead, in MRAFF, a magnetorheological fluid is mixed with abrasive particles and then dispersed in a nanomagnetic carrier. When a magnetic field is applied, the rheology of this fluid changes and facilitates the control of finishing forces.

Chawla et al. [68] developed a MAAFM setup to optimize the changes in surface roughness (∆*Ra*) and material removal rate (MMR) of an Al/SiC/B4C hybrid metal matrix composite. According to the results, MRR and ∆*Ra* significantly increased with an increase in extrusion pressure (51.83%, 101%), while with a decrease in mesh number, MRR and ∆*Ra* improved by 14.56% and 22.58%, respectively. With increased abrasive concentration, the roughness decreases by 8.18%. On the other hand, a rise of 13.5% in MRR resulted from an increase in magnetic flux density.

Further developments arrived at ultrasonic-assisted AFM (UAAFM) that integrates ultrasonic energy with AFM. In this finishing, ultrasonic vibration is applied to the pressurized abrasive flow before reaching the internal channel. In this way, it is possible to increase the interaction forces between the abrasive grains and the surface of the internal channel and, as a result, improve the efficiency of the finishing operation [69,70]. Choopani et al. [71] developed a new nano finishing for the internal surface of Al2024 tubes, called the ultrasonic assisted-rotational magnetorheological abrasive flow finishing (UA-RMRAFF) process, in such a way that the ultrasonic vibrations were perpendicular to the magnetorheological polishing (MRP) fluid flow direction. According to the results of experiments, UA-RMRAFF provided a uniform and fine surface finish without surface defects, such as mirrors up to 25.5 nm, as well as increased material removal. In another attempt by Dixit et al. [72], the rotating effect was combined with ultrasonic assistance and magnetic field assistance to the surface finishing of 3D-printed acrylonitrile butadiene styrene (ABS) and polylactic acid (PLA) parts. After optimizing the process parameters such as magnetic flux density, extrusion pressure, vibration intensity, and iron particle concentration as the abrasive particles, the maximum material removal of 26.62 mg and Δ*Ra* of 54.42% were achieved.

Through the comparison between different AFM, it can be concluded that the rheological characteristics of the abrasive media are critical. Thus, several researchers have been developing new abrasive media that can enhance the quality of AFM. Nonetheless, despite several efforts that have been made on AFM, several limitations still exist. For instance, blind holes are still challenging to be machined through AFM technology. Furthermore, fluid flow characteristics of the abrasive media are another limitation that makes the uniform finishing operation very difficult. Finally, contamination problems due to the abrasive particle’s embodiment onto the surface of the workpiece are another challenge in AFM.

### 3.2. Magnetic Abrasive Finishing (MAF)

**Figure 9:**
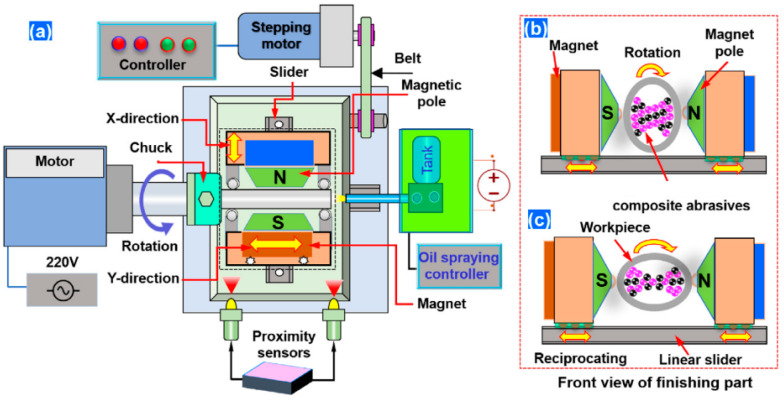
(**a**) Schematic illustration of MAF process, (**b**) front view of finishing process on span portion, and (**c**) on rise portion [76].

The second non-traditional finishing method is MAF, which has high precision. In this method, a magnetic field controls the finishing forces during the finishing operation [73]. This method was introduced first in the Soviet Union and was followed by many countries including the United States, Germany, Bulgaria, and Japan [74,75]. Initially, this method was developed to finish the cylindrical parts [56]. After that, in the 1990s, through a slight modification, MAF was adopted for finishing the internal surface of tubes [75]. Figure 9 shows the schematic of MAF for internal surfaces. As seen in this Figure, applying the current to the coil creates an electromagnetic field that accumulates the abrasive particles at the finishing area and works as a flexible brush. It is fascinating to highlight that the concept of MAF is similar to the AFM method, with the magnetic force generated between magnetic abrasive and N-S magnetic poles. It is interesting to note that, in some setups, it is also possible to vibrate the workpiece to increase the efficiency of the finishing operation. In fact, since material removal is performed using a magnetic field in this method, the risk of crack formation on the external surface of the workpiece is negligible, which can be the most significant advantage of this technology over conventional ones such as grinding, honing, or lapping.

To avoid the excessive frictional force of the particles on the internal surfaces, a lubricant such as oil is also fed into the channel during the finishing operation. Since in the MAF process, the finishing operation is carried out using an external electromagnetic field on the abrasive particles to guide them into the finishing area; the abrasive particles should have high magnetic susceptibility in addition to their abrasive characteristics. In this way, under the electromagnetic field, magnetic forces are formed and penetrate the abrasive particles onto the surface of the channel. For this reason, the abrasive particles in this method are conglomerates containing a highly magnetic-susceptible material, like iron, and hard abrasive material, like nano alumina. Magnetic abrasives, such as hard iron alloys, are usually used to finish soft or non-ferrous metals [77]. Instead, for a workpiece with a harder material, it is crucial to use a mixed-type abrasive consisting of SiC or Al_2_O_3_ with some ferromagnetic particles [78]. In addition, magnetic abrasives can be applied as a form of abrasives held in a ferromagnetic matrix formed by sintering, chemical, or other techniques [79,80]. According to its remarkable finishing effects on MAF, the sintered magnetic abrasive has been used more than the others [81,82]. Regarding its costly and complex process, mixing steel grit with SA is preferable.

**Figure 10:**
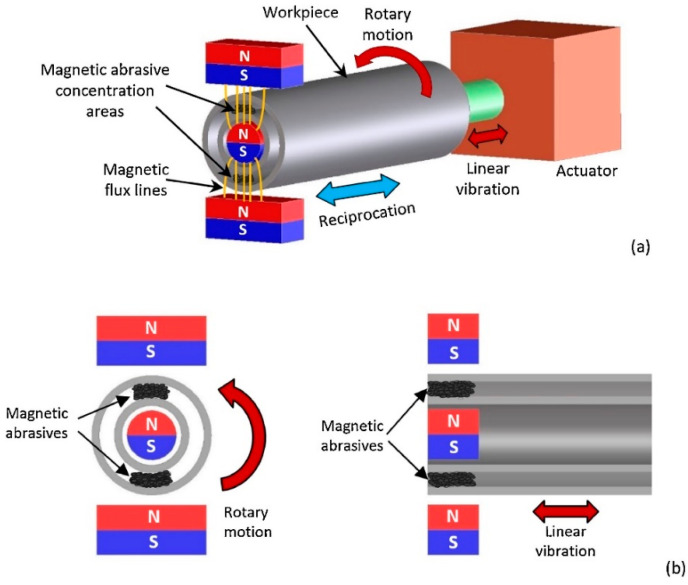
Schematic of the rotating-vibrating magnetic polishing method, (**a**) full view, (**b**) cross-sectional view [29].

After applying the electromagnetic field and penetration of abrasive particles into the internal surfaces, through the fast rotation of the sample or magnetic pole system it would be possible to create a motion between the abrasive particles and internal surfaces and, consequently, remove the material in the form of fine abrasion. It is well documented that this method can finish external and internal surfaces to obtain surface roughness down to a few nanometers. For instance, Zhang et al. [83] used MAF to finish the surfaces of AISI 316L parts produced via the L-PBF process. Their results confirmed that MAF improved the *Ra* of the surface up to 76%. Recently, Guo et al. [29] investigated the effect of the rotating-vibrating magnetic polishing method on the surface quality of the internal channels in the AM IN718 part (Figure 10). In fact, they integrated rotation and vibration magnetic polishing methods to increase the finishing process’s efficiency. However, from the configuration of this method shown in Figure 10, it seems this method for complex internal cooling channels has several limitations.

**Figure 11:**
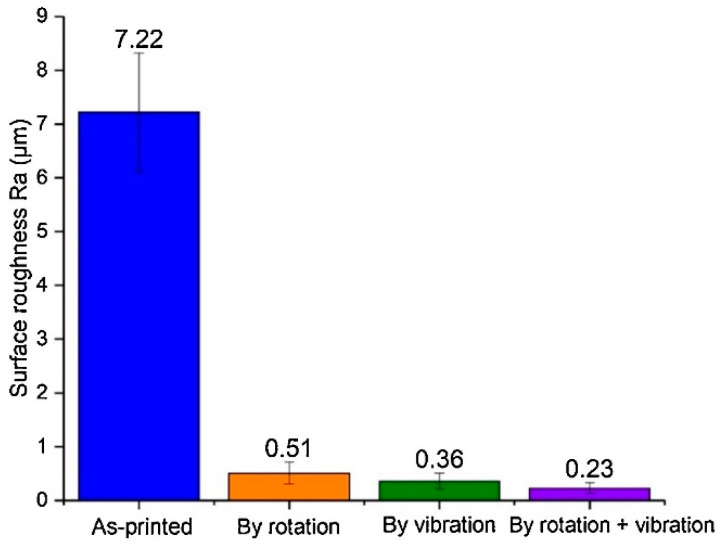
Surface roughness of internal channel in L-PBF IN718 part after different polishing methods [29].

They showed that this method reduces *Ra* from 7.22 μm to 0.51 μm, 0.36 μm, and 0.23 after polishing using rotation motion, vibration, and integration, respectively (Figure 11).

Saxena et al. [84] studied the influence of various process parameters, such as working gap, rotational speed, and machining time, on the MAF process of a mild steel cylindrical part. Their results show that the lowest surface roughness for this case (*Ra* equal to 1.49 μm) can be achieved using 35 mm, 430 rpm, and 2 min as the working gap, rotational speed, and machining time, respectively. In another work, Aizawa et al. [85] studied the internal surface finishing of a stainless sanitary tube using the MAF method. They revealed that using a correct concentration of magnetic fluid for 10 min finishing time, it would be possible to successfully reduce the internal surface in a stainless steel sanitary tube from 4 μm to 0.1 μm.

Zhu et al. [86] used spherical composite magnetic abrasive particles (MAPs). They investigated the effect of different parameters such as spindle speed, feed speed, and machining gap on the AlSi10Mg produced by the L-PBF technique. The MAF method helped to reduce surface roughness from 4–10 μm to ~10 nm and improved the surface quality.

**Figure 12:**
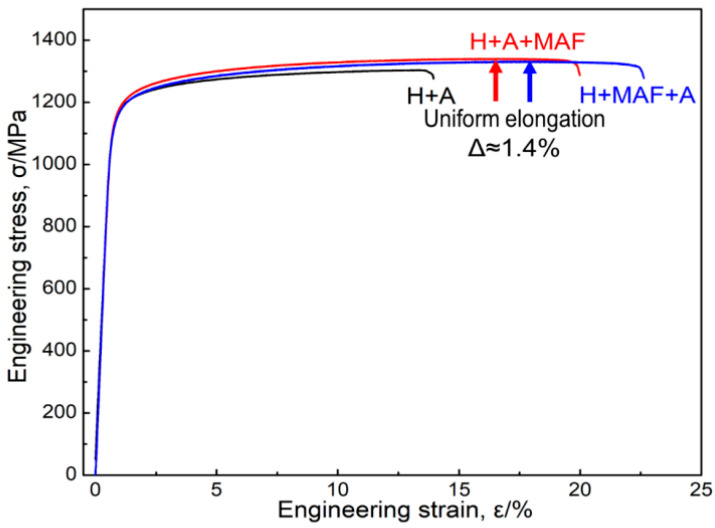
Stress–strain curves of the fully heat-treated sample with homogenization process (H) and aging process (A) (without the MAF process), sample H + A + MAF (with the MAF process after the full heat treatment), and sample H + MAF + A (with the MAF process between the homogenization and aging processes) [88].

In the two similar works by Zhao et al. and Li et al., the effect of MAF and heat treatment conditions on the microstructure of additively manufactured Inconel 718 super-alloys was studied [87,88]. The combination of the two treatments provided a reduction in surface roughness, refinement in grain size, and improvement in alloy elongation. The stress–strain properties of alloys with different processes are shown in Figure 12.

Another critical factor in this technology is the relative hardness of the component concerning the abrasive particles that determine the depth of cut obtained by the abrasive particles. As mentioned earlier, since in this technology the material removal is carried out by a magnetic field, it implies that the strength of the magnetic field, which is adjusted by the distance between the yoke and the part, plays a key role. The influence of this aspect would be more important in the case of bend tubes or internal channels where different finishing forces are required at inner and outer radii zones [89]. MAF’s time is another crucial factor that should be optimized carefully. The importance of this parameter comes back to the sequence of phenomena that occur during the process. It is reported that surface roughness is improved significantly in the first minutes of the MAF process, and beyond that cannot be improved. After this short period, only material removed from the surface takes place that might lose the dimensional accuracy.

Apart from the experimental investigation, several efforts based on simulation and modeling have been made on this technology [90,91]. For instance, Kim et al. [91] modeled and simulated the MAF process and concluded that the magnetic flux density in the air gap is greatly affected by the length of the air gap; magnetic flux density increases as the length decreases. Other internal surface finishing simulation results agree better with the experimental data for the low magnetic flux density than the high magnetic flux density one [81,92]. Wang et al. [93] used multi-abrasive particles in magnetic abrasive finishing for complex blind cavities using the discrete element method (DEM). The wall-ware mode and particle motion were simulated based on the particle size, speed of the magnetic pole, and processing clearance. The results obtained from the simulation were in good agreement with the experimental data. Surface roughness decreases by 89.3% after optimizing the process parameters.

Despite the successful finishing of various AM parts made of different materials such as AlSi10Mg, Ti6Al4V, Inconel 625, and Inconel 718 alloys, reaching 0.21 μm for *Ra*, it has been found that internal surface finishing of channels with a diameter less than 1 mm is still challenging [29,94,95]. Besides, the disadvantages of the MAF method are related to its necessity of using bonded abrasives, the part or magnet rotation, and the insertion of a tool inside the internal channels. Moreover, the limited list of materials that can be surface finished through the MAF method is another significant limitation of this technology. In fact, through this technology, the internal surface finishing on ferromagnetic materials such as Ni and Co alloys is negligible. This limitation comes back to the nature of these alloys that become magnetized upon applying a magnetic field and then strongly absorb the abrasive particles. Due to this absorption of particles on the surface, their relative motion would be blocked, and, thus, no material removal is taken place. Another limitation in the technology is related to the shape and geometry of the internal features. For instance, in the case of fine and protuberances, since the abrasive particles cannot navigate around these futures, MAF technology has very low efficiency.

### 3.3. Fluidized Bed Machining (FBM)

**Figure 13:**
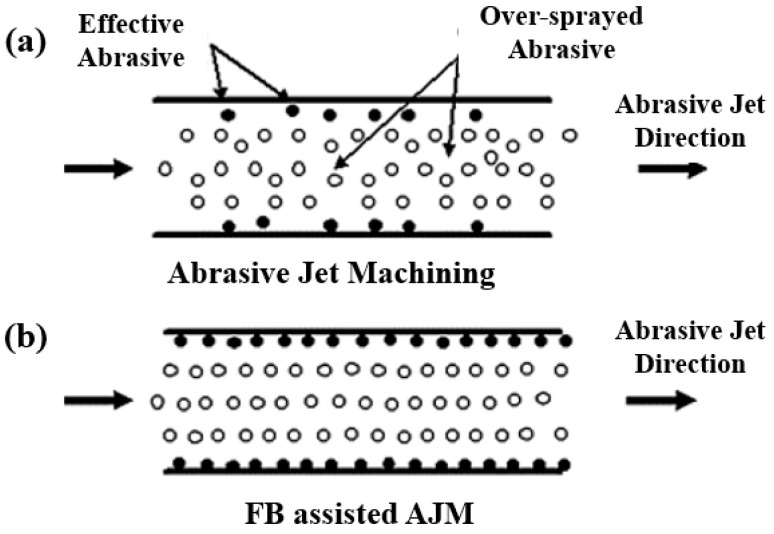
Schematic of (**a**) abrasive jet machining, (**b**) fluid bed abrasive jet machining [99].

FBM is an advanced surface finishing method that uses fluid bed hydrodynamics to finish the surface [96]. This method is used to finish both external and internal surfaces. In FBM, a fluidized bed is formed when a bed of solid abrasive particles is adjusted under fluid flow. In this way, the bed of abrasive particles behaves like a liquid and performs the finishing operation [97]. However, further developments introduced fluidized bed abrasive jet machining (FB-AJM) specifically for internal surface finishing [98]. FB-AJM seems to be the most competitive with respect to former technologies as it requires shorter start-up times as well as lower investment and running costs. In this method, a mixture of working fluid and abrasive particles is jetted inside the internal channels. In this system, two fluidized beds are combined with abrasive particles and injected into the internal channel. Figure 13 compares the AJM and fluidized bed AJM.

In addition to the abrasive and workpiece characteristics, fluidization property is the critical factor that defines the capability and efficiency of the process in both FBM and FB-AJM processes. It is reported that if the feeding of abrasives into the internal channel can be automatically reversed through the fluidized beds, a uniform surface can be obtained afterward. The basic machining is done through jetting, whereas the fluid beds increase the machining efficiency, reproducibility, and surface quality. For instance, it is reported that at a minimum fluidization region, since the weight of abrasive particles counterbalances the hydrodynamic push, the abrasive bed starts to transit between a fixed bed and a bubbling bed [100]. By increasing the flow rate, the size of bubbles grows and as soon as the abrasive bed reaches a pneumatic region, the bubbles disappear. In this region, the impact speed increases significantly and can arrive at 50 m/s, resulting in higher MRR. However, higher impact speeds also increase the indentation depths, resulting in inaccurate surface finishes.

Overall, these two processes have a high potential for fine finishing operations in such a way that through these processes it would be possible to finish the surface to lower than 0.4 μm. For example, FBM has been used for the surface finishing of narrow and long tubes made of aluminum [101] and stainless steel parts [102]. In contrast, FB-AJM has been employed to perform a high-quality surface finishing on difficult-to-machine nickel alloys [98]. Barletta et al. [101] performed the finishing operation inside the internal channels of AISI 316L stainless steel employing an abrasive jet system and two fluid beds. One of the main advantages reported for this machining process is its capability to finish long channels. For example, Barletta et al. [97] surface finished AA 6082 T6 internal channel with a diameter of 10 mm and length of 350 mm. Using this technique, they reduced *Rz*, which is the arithmetic height of the profile, from 40 μm to 5–7.5 μm after just four machining cycles. Another advantage of FBM and FB-AJM is their independence from the initial surface roughness, and the finishing operation time depends on the abrasive size. Another critical parameter is workpiece rotation speed and abrasive impact velocity. Nevertheless, it should be underlined that these two technologies face several challenges, such as abrasive contamination of soft workpieces, difficulty in the surface finishing of the linear channels, and longer diameters [13]. Overall, according to the efforts undertaken in adopting PBF technology for the surface finishing of internal channels, surface finishing of internal channels with complex bends using PBF is very challenging.

**Figure 14:**
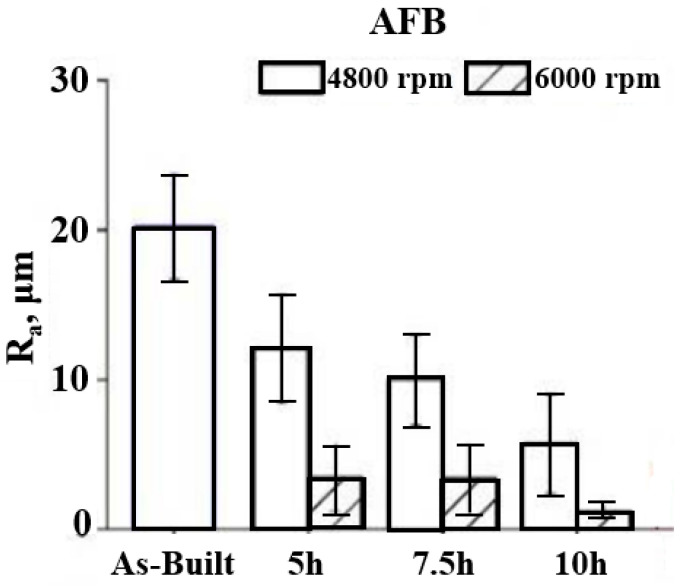
Average surface roughness of the AFB-treated samples for different speeds of particles and various treatment times [103].

Atzeni et al. [103] studied the influence of the abrasive fluidized bed method on the fatigue life and roughness of Ti-6Al-4V alloys manufactured by the electron beam melting method. As can be seen in Figure 14, a smoother surface was obtained at the higher rotational speed of abrasive particles (6000 RPM), and surface roughness increased with the treatment times. However, on the other hand, lower rotational speed increased the fatigue life (257,793 cycles).

**Figure 15:**
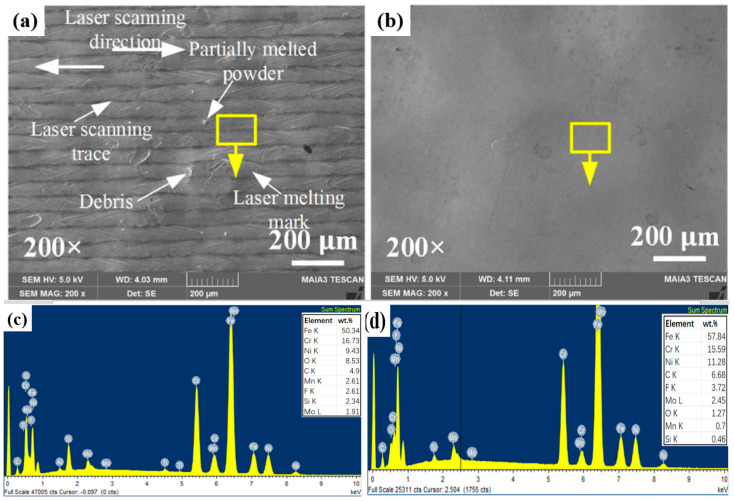
SEM photographs of the top surfaces of 316L stainless steel (**a**) before and (**b**) after MJP polishing. Composition analysis (**c**) before and (**d**) after MJP polishing [105].

Sahu et al. [104] worked on the machinability of K-90 alumina ceramic by the fluidized bed abrasive jet machining (FB-AJM) technique. The results of the orthogonal array design of experiments confirmed that although MRR increases directly with pressure and standoff distance, it showed an increment and then a reduction with an increase in the abrasive mesh size. More recently, Wang et al. [105] proposed a multi-jet polishing (MJP) method to finish additively manufactured 316L stainless steel. Figure 15 shows the top surface topography before and after polishing. Some laser melting marks and debris could be observed on the surface before polishing, but after MJP the laser melting marks, debris, partially melted powders, and cracks were eliminated on the surface, revealing that MJP can successfully smoothen the PBF 316L SS (reduction of surface roughness from 2.269 μm to 0.030 μm). According to the energy dispersive X-ray spectroscopy (EDS), no obvious change could be found on the surface, suggesting that MJP had no effect on the chemical composition of the PBF 316L SS. The only difference was related to the reduction of the oxygen element percentage. Laser melting produces oxide layers on the surface because of oxidization at high temperatures. These oxide layers can be removed after polishing, resulting in the reduction of the oxide element.

### 3.4. Cavitation Abrasive Finishing (CAF)

Cavitation abrasive finishing has great potential for the surface finishing of AM parts [106,107]. In this technology, ultrasonic and hydrodynamic methods are used to create cavitation. The difference between these two methods is their application. The simplest method, which is the ultrasonic one, is usually used for the external surfaces. In contrast, the hydrodynamic method, which is more flexible, is employed for the finishing of both external and complex internal channels. The working principle of CAF involves the controlled generation and collapse of cavitation bubbles in a liquid medium to achieve the surface finishing of components. Cavitation refers to the formation of vapor-filled bubbles or voids in a liquid when subjected to rapid changes in pressure. In CAF, the process typically begins by immersing the workpiece or component in a liquid, often referred to as the finishing medium. This liquid is chosen based on its physical properties and compatibility with the material being processed. Common examples include water, oil, and specialized chemical solutions [108]. Next, ultrasonic energy is introduced into the liquid medium through the use of transducers or ultrasonic horns. These devices generate high-frequency mechanical vibrations, typically in the range of 20 kHz to several hundred kHz [109]. These vibrations propagate through the liquid, creating alternating areas of high- and low-pressure regions. The alternating pressure regions induce the formation and subsequent collapse of microscopic cavitation bubbles near the surface of the workpiece. During the expansion phase, the low-pressure regions cause tiny vapor-filled bubbles to form. These bubbles grow in size until they reach a critical size, at which point they rapidly collapse during the compression phase. The collapse of the cavitation bubbles produces localized high temperatures and pressures, generating intense shockwaves and microjets in the surrounding liquid. These phenomena result in the removal of material from the surface of the workpiece through a combination of mechanical impact, erosion, and micro-scale abrasion. The combination of the mechanical impact and fluid dynamics associated with cavitation allows for the removal of surface irregularities, such as burrs, roughness, and contaminants. It can also facilitate the smoothening and polishing of surfaces, resulting in improved surface finish and texture [28,110]. The CAF process parameters, such as the ultrasonic power, frequency, exposure time, and liquid composition, can be adjusted to control the intensity and distribution of cavitation, thereby influencing the material removal rate and the desired surface characteristics [111].

**Figure 16:**
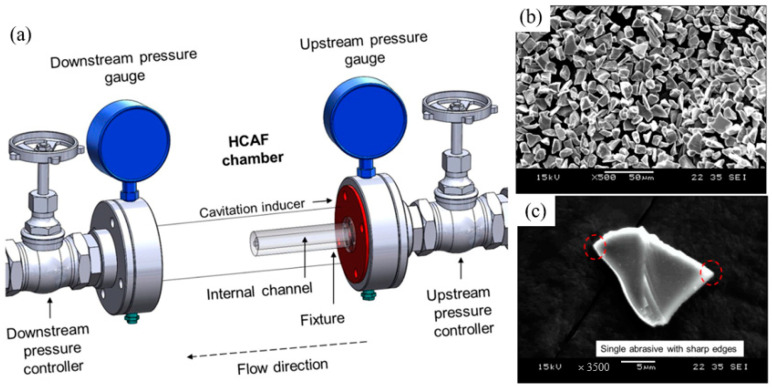
(**a**) Details of the HCAF chamber, (**b**) SEM image of the SiC abrasive used in the finishing operation, (**c**) Single abrasive with sharp edges [28].

**Figure 17:**
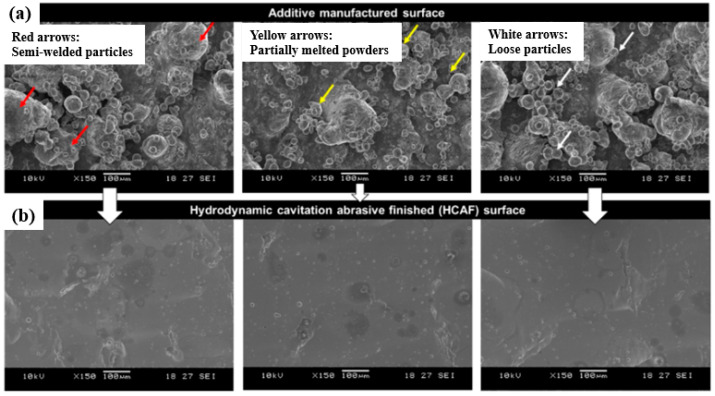
SEM micrograph from the surface of the (**a**) as-built internal channel and (**b**) after internal surface finishing using the HCAF process [28].

For the first time, hydrodynamic cavitation abrasive finishing (HCAF) was introduced by Nagalingam et al. as a reliable method for the surface finishing of the internal channels [112]. Yeo and Nagalingam [113] extended the HCAF method as multi jet hydrodynamic cavitation abrasive finishing (MJ-HCAF). The fundamental of these two technologies is based on using generated cavitation bubbles for the finishing operation. These two technologies usually add micro-particles in the multiphase flow to accelerate the finishing process. As presented in Figure 16a, one of the first internal channels, surface finished using these technologies and less than 1% abrasive, was made of AlSi10Mg alloy produced by the L-PBF process. Figure 16b,c shows the sharp-edged silicon carbide abrasive. The red circles in Figure 16c are relevant to the sharp edges of a single abrasive [28]. The as-built surface roughness of those internal channels was very high (~*Ra* = 20 µm, and *Rz* > 100 µm). They found that by using the HCAF process, it would be possible to improve the surface texture (up to 90%) and, after a 150 min finishing operation, reach a surface with a very low surface roughness (~*Ra* = 3 µm, and *Rz* = 30 µm) (Figure 17).

**Figure 18:**
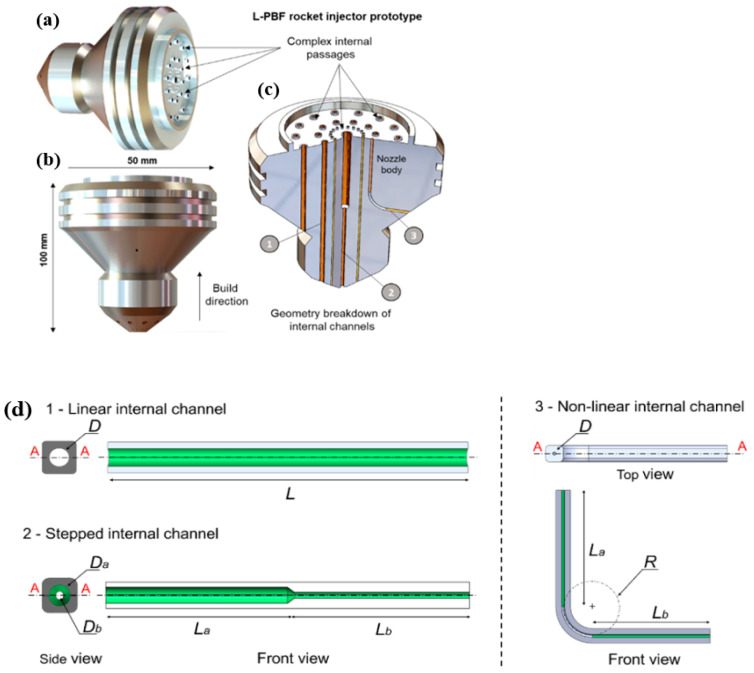
(**a**–**c**) L-PBF rocket injector prototype, (**d**) internal channels for surface finishing; (1) linear, (2) stepped, and (3) non-linear channels [27].

**Figure 19:**
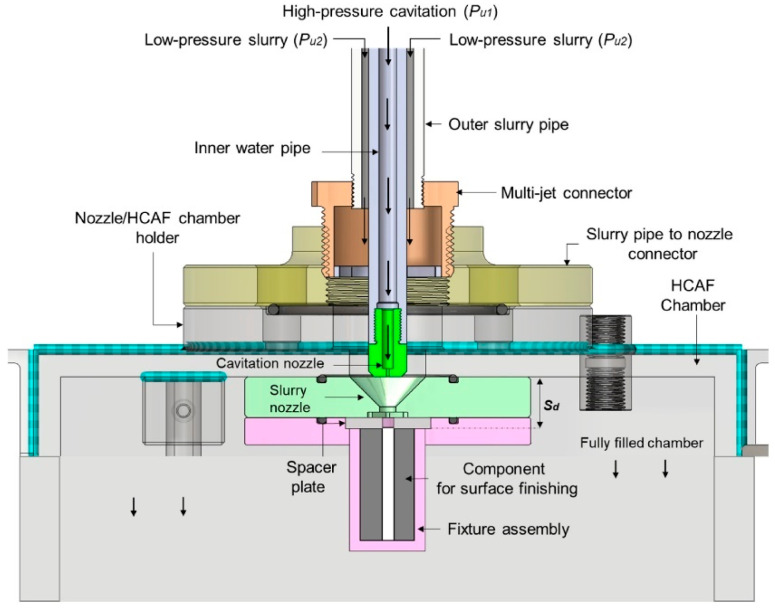
Details of MJ-HCAF process used for the surface finishing of Inconel 625 internal channels [28].

**Figure 20:**
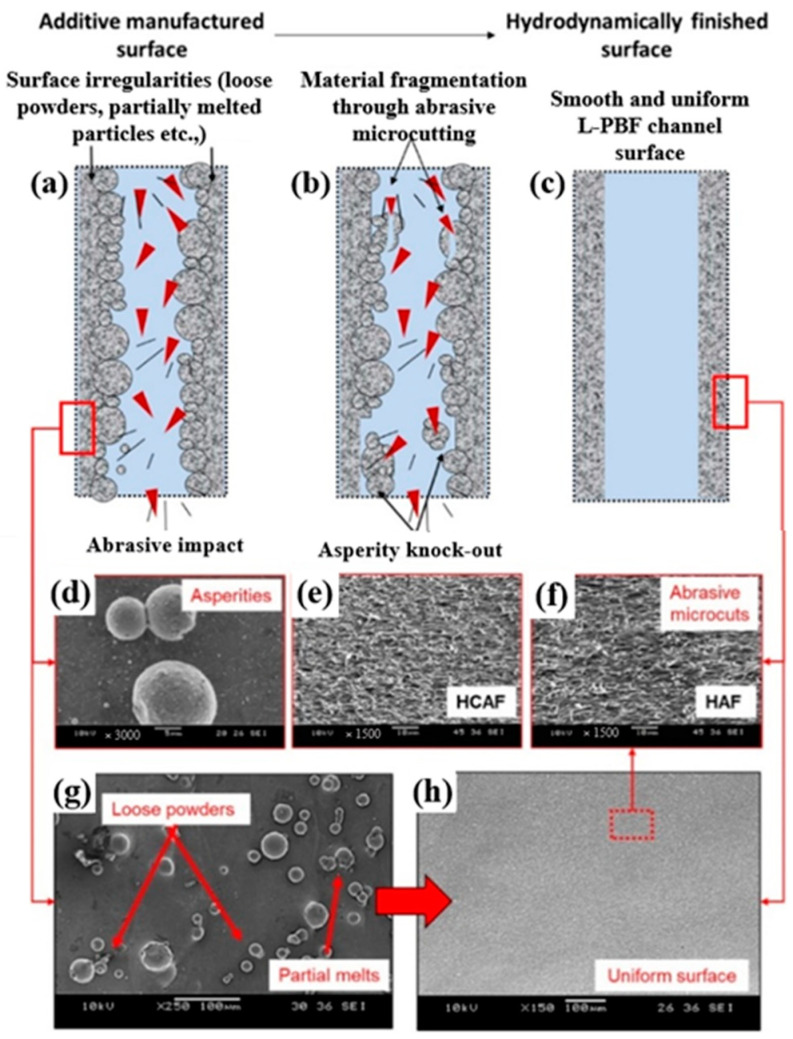
Influence of micro-abrasives in the surface finishing of the AM channels, (**a**–**c**) steps of materials removal, (**d**) as-built surface, (**e**) hydrodynamic abrasive finished surface, (**f**) hydrodynamic cavitation abrasive finished surface showing abrasive micro cuts, (**g**) as-built surface morphology with irregularities, and (**h**) smooth and uniform texture after surface finishing [28].

In another work, Nagalingam and Yeo [27] studied the surface finishing of L-PBF Inconel 625 complex internal channels (Figure 18) using the MJ-HCAF process (Figure 19). Linear internal channels refer to straight and uninterrupted pathways or passages within a component that allow the flow of fluids, gases, or other substances. In stepped internal channels, the internal pathways or passages within a component, such as a rocket injector, fuel nozzle, or cooling channel, have step-like variations in their dimensions. Unlike linear channels, non-linear internal channels deviate from a straight or linear geometry and exhibit curvatures, bends, twists, or irregular shapes. Their outcomes revealed that using the MJ-HCAF method together with an abrasive concentration of <1% for 15 min to finish the surface of Inconel 625 internal channels resulted in 60–90% surface improvement (*Ra*, Sa < 1 μm and *Rz* < 20 μm). They showed that when the micro-abrasives are used in the working fluid, the random surface asperities are removed gradually, resulting in surface erosion (Figure 20). Due to surface erosion, a homogenized surface texture is formed in the internal channels.

In a more recent paper, Nagalingam et al. [110] developed a novel multi-jet hydrodynamic cavitation-based finishing (MJ-HCAF) method to smooth the surface of the L-PBF Inconel 625 internal surfaces. The primary fluid with abrasives particles ≤1.0% led to the ~85% improvement in surface quality (Sa ≤ 0.5 µm and Sz ≤ 10 µm).

**Figure 21:**
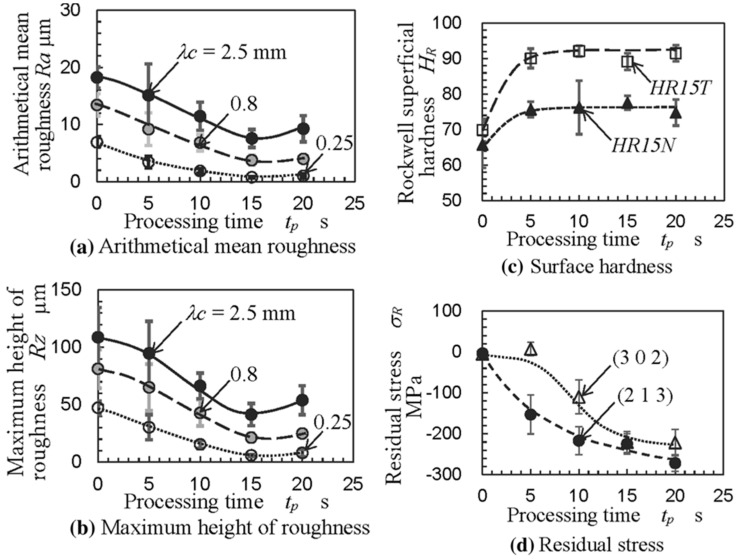
(**a**,**b**) Surface roughness parameters, (**c**) surface hardness and (**d**) residual surface stress of EB-PBF Ti-6Al-4V parts before and after surface finishing [114].

It is interesting to note that the HCAF process not only improves the surface roughness of the internal channels but also can enhance the microhardness and microstructure of the internal channels. For instance, Soyama and Sanders [114] found that by using the HCAF surface finishing process, it would be possible to improve the compressive residual stress of the parts, which can enhance the fatigue life of the EB-PBF Ti-6Al-4V parts (Figure 21).

The essential features investigated so far for the internal surface finishing of AM part are geometry, length, diameter bend or curvature, tapered, varied cross-section, and branches. Therefore, it can be concluded that to achieve an appropriate surface finish using the HCAF process, it is necessary to consider all the aforementioned factors and find the best process parameters.

### 3.5. Electrochemical Finishing (ECF)

Electrochemical finishing processes are chemical energy-based processes that are not related to the mechanical properties of the materials [115,116] and make the surfaces with superior corrosion resistance and stress relief, as well as making them hygienically clean [117]. This method removes oxide films on the strong passive materials using aggressive and hazardous chemical species added to viscous, nonaqueous and/or highly acidic electrolytes. For example, the surface of enormously passive metals like niobium and nitinol alloys is de-passivated by adding hydrofluoric acid and fluoride salts to the traditional electrolytes [118]. The most important benefit of the electrochemical process is excellent compatibility with a wide range of other processes and different energy sources [119,120,121,122,123,124]. In addition, the requirement for less tooling than other techniques like AFM or MAF is another excellent advantage of this method. It is worth noting that electrochemical finishing is the only technique suitable for removing the materials of the free form and open porous lattice structure [13].

The thickness loss due to the electrochemically polishing is as high as around 80 μm [125,126]. As a result, polished parts are considered to have weak dimensional integrity, which is a significant disadvantage. Electrochemical polishing is also associated with additional concerns, such as a large setup for big components, high amounts of chemical materials, high voltage, and safety precautions [13].

Some research has been done to define the electrolyte types and process specifications of electrochemical deburring, polishing, and boring [127,128,129] and use electrochemical polishing in biomedical applications to achieve a roughness of 0.09 µm in titanium orifice [130]. More recently, researchers were focused on electrochemical polishing and finishing of stainless steel, tungsten carbide, and 6061 Al/Al_2_O_3_ composite workpieces [131,132,133].

**Figure 22:**
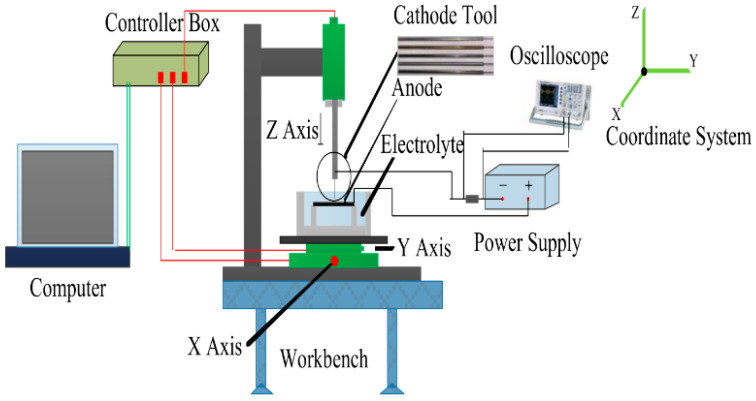
Schematic diagram of electrochemical machining [139].

In electrochemical processes, the coupled workpieces and tool act as anode and cathode and cause a sort of differential dissolution to remove the materials from the surface. This condition needs an appropriate electrolyte and a high current with low DC voltage. Viscose acid is typically used as an electrolyte [134]. The anode in this process has a passive film formed of metallic oxide to cover the lower peaks while the higher peaks remain uncovered. The voltage is constant during the process, so the current densities differ for different uncovered peaks based on their heights [135,136,137,138]. The schematic diagram of electrochemical surface machining is illustrated in Figure 22.

Surface smoothing occurs by anodic levelling (macro-smoothing) and anodic etching (micro-smoothing) in ECP/ECF [116]. In macro-smoothing, dissolution differences between the peaks and valleys cause the smoothing, whereas in the latter the process is the crystallographic etching controlled by surface defects [140]. The power supply in the ECF process controls the volume and rate of material removal [116,134,135,141].

A combination of electrochemical machining (ECM) and MAF is a more productive process called electrochemical magnetic abrasive finishing (EMAF). This process has higher efficiency with respect to the single MAF or ECM, and the abrasives could be protected [142,143]. This combination especially shows its benefit in the case of soft and non-ferromagnetic materials like Al6061, which are difficult to process with MAF because of their debris that is not easily absorbed by the magnetic abrasive brush [142]. It consists of a primary coil, spindle turning, workpiece fixture, and auxiliary coil. The composite tool was separated as an electrode and magnetic pole using different non-ferromagnetic brass materials and ferromagnetic soft iron. However, except for the soft materials, it is beneficial for hard alloy materials to get high efficiency of machining and high accuracy. Sun et al. [144] improved the finishing efficiency of stainless steel of SUS304 by higher than 75% and obtained less surface roughness by the EMAF process than the traditional MAF technique. The finding of experiments showed that the roughness reduced from 0.178 μm to 0.03 μm after the EMAF process.

Another type of electrochemical finishing process is ultrasonic vibration electrochemical finishing. The ultrasonic vibration process primarily aims to effectively discharge electrolytes and by-products in ECF/ECP. Experimental results show that ultrasonic vibrations can give 21–44% improvement in surface finish, depending upon the process variants and conditions used. The use of ultrasonic energy began in 1927 to produce holes in a glass bar. Later, the process improved for welding, metallurgy, and cleaning processes. Likewise, it has been used in electrochemical finishing processes to enhance surface finish [145,146,147].

**Figure 23:**
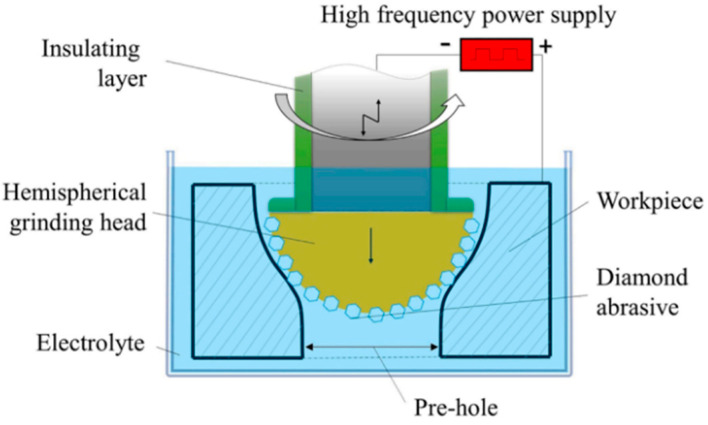
Schematic of the UAECDG machine [149].

**Figure 24:**
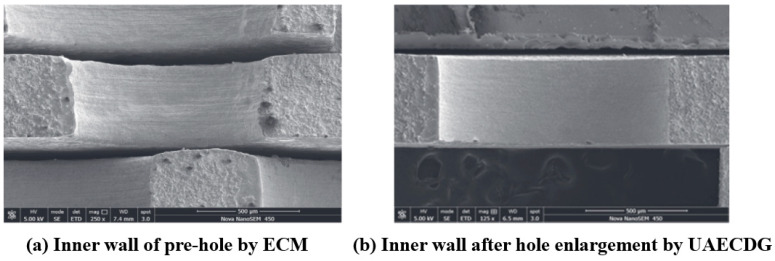
The inner wall of a hole before and after UAECDG treatment [148].

Zhu et al. [148] combined ultrasonic electrochemical machining and mechanical drill grinding to propose an ultrasonic-assisted electrochemical drill grinding (UAECDG) schematically presented in Figure 23. They studied the effect of the proposed technique on the machining accuracy and the enlarging of 304 stainless steel holes through both experimental and simulation methods. UAECDG was able to machine small holes with a surface roughness of 0.31 μm, a diameter of 1.1, and a taper < 0.6 degrees. An SEM analysis of the inner wall of a small hole can be observed after UAECDG treatment in Figure 24. In similar research by Shu et al. [149], UAECDG was applied on L-PBF printed Hastelloy X holes to machine the holes with a 1215 μm final diameter and roughness of 0.446 μm. As a result, a significant improvement in machining accuracy can be achieved by using UAECDM technology for metal AM parts with small hole structures.

**Figure 25:**
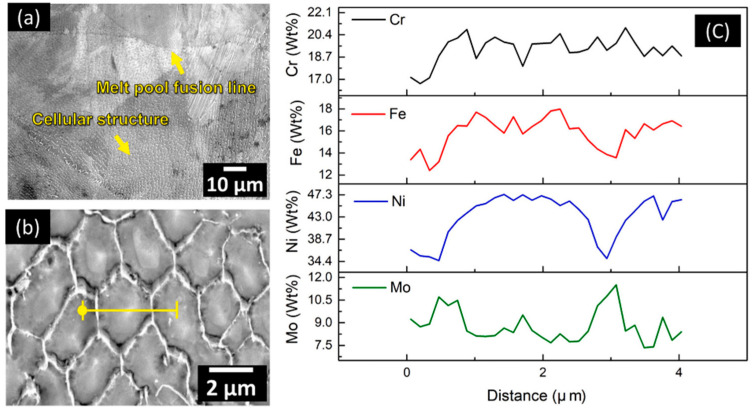
(**a**) SEM image of the 5 min electropolished Hastelloy X; (**b**) higher magnification SEM image of the cellular structure, with the yellow line corresponding to the start and the end position for the EDS analysis; (**c**) EDS analysis showing the wt% variation of the Cr, Fe, Ni, and Mo along the scanning direction [150].

Jiang et al. [150] investigated the surface roughness of additively manufactured superalloy Hastelloy X (HX) using electropolishing in a mixture of choline chloride and ethylene glycol. The internal surface quality of the angled tube L-PBF HX with a diameter of 3 mm was successfully enhanced during electropolishing. Figure 25 shows a fine dendritic and cellular morphology with melt pool fusion lines after electropolishing of the HX alloy. The SEM-EDS scanning analysis across the cellular structure suggested that the cell boundary is mainly rich in Mo, whilst the core of the cell is rich in Ni and Fe. Based on the EDS results, Ni and Fe were more favorably dissolved in choline chloride and ethylene glycol electrolyte compared to Mo and Cr.

**Figure 26:**
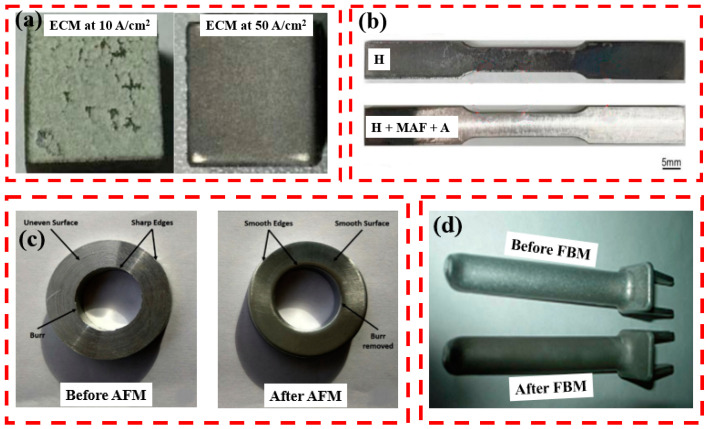
(**a**) Surface finish of Ti60 alloy after ECM at different current densities [151], (**b**) surface morphologies of the Inconel 718 superalloys with a sequence of H (homogenization) + MAF + A (aging) [88], (**c**) cylindrical aluminum workpiece before and after AFM [152], and (**d**) surface finished AA 2024 O alloy after FBM [97].

Figure 26 shows the types of samples that surface finished using different techniques. Figure 26a shows the high-temperature Ti60 alloy machined at different current densities in the 10 wt.% NaCl electrolyte and 40 °C temperature. The machined sample was very smooth under a current density of 50 A/cm^2^. By contrast, the workpiece surface showed uneven surface and pitting corrosion when the current density was 10 A/cm^2^. To ensure the quality, efficiency, and stability of ECMs, a high current density of more than 20 A/cm^2^ is recommended [151]. The homogenized (H) printed surface of Inconel 718 superalloys, followed by the MAF and ageing (A) process, is shown in Figure 26b. The sample showed a shiny surface after MAF, indicating that MAF was effective in polishing. Figure 26c shows the ring-shaped cylindrical aluminum alloy workpiece before and after AFM. AFM completely removed all the burrs, sharp edges, and uneven surfaces, creating a smooth surface. In Figure 26d, FBM led to smoothing the concave shape part in AA 2024 O alloy (surface darkening was due to the employment of black alumina as an abrasive).

Table 2 presents a summary of non-traditional surface finishing for additively manufactured materials.

## 4. Theoretical Framework

### Preliminary and Scope

Surface finishing has always been a central issue in engineering, and researchers have often attempted to provide tools, sometimes very sophisticated, to develop the processes dynamically. This stream of research has been accelerating for the past decade, perhaps due to the increasing levels of manufacturing and its complexity, such as additive manufacturing. From a managerial point of view, advanced manufacturing, although providing many benefits to the development of products, has brought more demands on surface finishing. Regarding these demands, several methods have been developed in five primary categories, which are abrasive flow finishing (AFF), magnetic abrasive finishing (MAF), abrasive fluidized bed machining (AFB), cavitation abrasive finishing (CAF), and electrochemical finishing (ECF).

Three key elements have characterized the focus of this review:

(I) The review addresses only the recent advances in process modeling. This focus distinguishes it from most of the contributions in the literature from the timescale perspective;
(II) The review covers different methods in surface finishing, while the other reviews focused on each one separately;
(III) The outcome of the models is considered here but with a comparison among all five so-called techniques.

## 5. A Framework for Literature Review

This section is structured in a detailed survey of models to study the recent advances in the internal surface finishing of complex-shaped components for good orientation in further steps. Our review is accomplished through the six critical issues on the internal surface finishing: component materials and shape, apparatus requirements, roughness change, limitation, internal surface accessibility, and post/pre-processing conditions. Table 3 lists the literature base on so-called issues compactly. Hence, this subsection aims to give implicit knowledge of the recent modeling trends, focusing on how researchers attempted to cope with internal surface finishing challenges.

## 6. Criteria Analysis and Discussion

In this section, different methods for surface finishing are analyzed based on the seven most critical factors and their accordance values. In this regard, each technique has its score for post-processing and pre-processing, and the scoring in Table 4 obtains the final decision about the combined method. Each factor has a score between one and five. Furthermore, in accordance with the different values of the elements, they have various credits in weight percentage among the others for obtaining the final score and evaluation of each method.

Seven different criteria are used to shape the comparison:

- Roughness improvement: Regarding the primary goal of surface finishing, material removal and roughness improvement are among the most important factors in all considerations. The roughness improvement score is 20% of the total.
- Internal surface finishing: Inevitably, accessibility for internal surface finishing is important for industrial components. Internal surface finishing score is 10% of the total. It should be noted that roughness improvement and internal surface finishing are both related to surface quality, but they differ in their focus and methods. Roughness improvement is primarily concerned with reducing the surface roughness of a material to achieve a smooth and uniform surface finish, while internal surface finishing is concerned with improving the quality and cleanliness of the internal surface of a material to optimize its performance and functionality.
- Size of components: In industries, parts are made in different sizes, and all machines and tools must be more flexible for different scales. The size of the components score is 10% of the total.
- Complex-shaped components: Surface finishing in complex-shaped workpieces is needed by the state-of-the-art finishing methods. Complex-shaped components score is 20% of the total.
- Possibility of finishing the Al and Ti products: Nowadays, Al and Ti components are expanding, and it seems essential to consider their consequences for surface finishing. The possibility of finishing the Al and Ti products score is 10% of the total.
- Cost of operation: Cost is an important index in all engineering activities. Costs of operation can be estimated by the features and complexities of the apparatus for each specific method. The cost of operation score is 10% of the total.
- Post- and pre-processing scores: For the best possible hybrid system, defining the best pre-processing and post-processing methods is crucial. In this regard, all methods are ranked as their ability for post- and pre-processing, and each of these numbers is influenced by the related final score for the post- and pre-processing, respectively.
- Post- and pre-processing scores have percentages of 20% of the total separately.

Table 4 shows the scores of the methods for different factors and concludes the final scores for pre-processing and post-processing surface finishing.

## 7. Future Directions

The measurement of hidden surface roughness will be challenging and require a custom solution for each AM component. Furthermore, in order to enhance the functionalities of AM components, surface finishing activities will also include applying various coatings on hidden and internal surfaces. A new frontier of research is emerging due to the unique geometry of AM components for targeted application areas. For example, electroless nickel coating processes [167] and atomic layer deposition (ALD) [168] can coat complex and three-dimensional structures uniformly, including hidden surfaces. The work on the coating area of surface-finished AM components is a new frontier of research and is vast in scope.

The use of non-contact or non-destructive methods for surface finishing of additively manufactured metallic components is another exciting future direction. These methods could include techniques such as plasma polishing or ultrasonic vibration, which could potentially offer advantages such as greater precision, reduced material removal, and faster processing times.

Future research could also investigate the potential for combining multiple finishing processes to achieve a more optimized surface finish. For example, a combination of mechanical and chemical finishing processes may be more effective at achieving a specific surface finish than either method used alone.

## 8. Concluding Remarks

(1) Since AM with poor surface finishing will fail prematurely in corrosive environments and cyclic loading, their internal surface finishing is the bottleneck preventing innovatively designed components from being applied in the intended environments.
(2) All non-traditional finishing processes discussed in this review have been successfully applied to a wide range of additively manufactured materials with low values of surface roughness. Nevertheless, based on the literature, ECM and CAF have shown an average reduction in roughness percentage of around 60–70%, but an improvement of more than 90% has been obtained for AFM and AFB.
(3) Both interior and exterior surfaces of AM metals can be polished with abrasive processes, which can be applied in rigid forms or in fluidized media. Abrasive finishing techniques lead to the reduction of surface roughness and create beneficial compressive residual stresses. In order to polish AM metals with common shapes, abrasive particles can be combined with magnetized particles added to ultrasonic cavitation.
(4) The choice of the most applicable internal surface finishing method for AM materials will depend on the specific requirements of the part and the desired end-use application. For example, the ECP process is the most promising for complex geometries and porous lattice structures.
(5) Through various reviewed methods and techniques, it has been found that the suitable combination of further studies could be EMAF for pre-processing and fluidized beds to a FB-AJM for post-processing.
(6) This paper provides a valuable resource for researchers and practitioners in AM, highlighting the importance of internal surface finish and the various methods and innovations available to achieve it.
(7) Despite many advantages offered by different advanced finishing processes, there are still limitations like size-limited processes, expensive operation, difficult finishing complex-shape components, and time-consuming processes, especially for large workpieces.

## Figures and Tables

**Table 1:** As-built surface roughness of metallic parts produced using different PBF machines.

Company	Process	Machine	Surface Roughness, *Ra* (μm)	Refs.
EOS GmbH	L-PBF	EOS M80, M100, M290, M300-4, M400, M400-4	9–50	[34]
SLM Solution	L-PBF	SLM 125, 280, 500, 800	8–17	[35]
Arcam EBM	EBM	A2	5.6	[36]
Arcam EBM	EBM	A2X, Q10 Plus, Q20 Plus, Spectra L and H	Horizontal: 19–30 Vertical: 24–39	[37]

**Table 2:** A summary of non-traditional surface finishing for additively manufactured material.

Alloy/AM Technique	Internal Surface Finishing Technique	Mechanism of Material Removal	Advantages/ Limitations of the Finishing Process	Reduction Percentage of *Ra*	Refs.
Al and Ti grills/L-PBF	AFM	Through a pressurized flow of viscoelastic material loaded with abrasives, the material is removed from the internal surface of the workpieces.	Achievement of a high level of surface finish/Time-consuming process, especially for large workpieces, uneven finishing and contamination problems.	More than 90%	[153]
AlSi10Mg/L-PBF	HCAF	Using high-velocity liquid jets that pass through a cavitating zone generates microbubbles that implode near the surface of the workpiece and remove material through erosion, abrasion, and fatigue.	Uniform surface finish/Difficult to remove material from harder materials due to the microbubbles.	More than 90%	[28,86,154]
	AFB	Using a fluidized bed of abrasive particles suspended in a high-velocity air stream impinges on the workpiece surface, removing material through a combination of impact, erosion, and abrasion.	Finishing large and irregularly shaped/Difficult to control the flow of the abrasive particles due to the high-speed impacts.	More than 90%	[155]
Inconel 625/L-PBF	MJ-HCAF	Cavitation erosion and abrasive ploughing.	High material removal rate/Difficult to control the distribution of the abrasive particles.	60–90%	[27]
	UCAF	Using ultrasonic waves creates cavitation bubbles in a liquid abrasive slurry, collapsing near the workpiece’s surface and removing material through erosion, abrasion, and fatigue.	Workpiece of most materials/Limitations in ultrasonic wave propagation.	40%	[32]
Inconel 718/L-PBF	MAF	The relative motion between the internal workpiece surface and magnetic abrasive cluster removes material.	Suitable for non-ferromagnetic materials/Difficult to remove ferromagnetic materials.	85%	[29]
304 stainless steel/L-PBF	ECM	The use of an electrolyte and an electric current to dissolve the workpiece material and create the desired shape.	It can work on difficult-to-machine materials, need to not so complicated tools/Relatively slow process, It can be changed in environmental variables.	55%	[156]
	Electrochemical mechanical polishing (ECMP)	Electrochemical reactions and mechanical abrasion.		70%	[157]
316L stainless steel/L-PBF	ECM	The use of an electrolyte and an electric current to dissolve the workpiece material and create the desired shape.	It can work on difficult-to-machine materials, need to not so complicated tools/Relatively slow process, It can be changed in environmental variables	60%	[158]
	Electropolishing	Dissolution of the metal resulting in the reduction of thickness.	It can remove surface imperfections, such as burrs and scratches, without affecting the underlying material; suitable for complex shapes and internal surfaces, which can be challenging to finish with other methods/Expensive; it can be changed in environmental variables.	80%	[159]
	Magnetically driven internal finishing (MDIF)	The use of magnetic fields to drive abrasive particles toward the materials’ removing.	Finishing the complex internal surfaces and highly controlled and precise process/The magnetic field strength and the abrasive particle size can affect the process.	More than 90%	[160]
	Multi-jet polishing (MJP)	Using high-velocity liquid jets impacts the surface of the workpiece and removes material through erosion.	Finishing the complex surfaces/Difficult to remove large amounts of material or to work on harder materials.	More than 90%	[105]

**Table 3:** Expanded view of the literature review.

Ref.	Method	Component Shape-Materials	Apparatus Requirement	Roughness Change (µm)	Limitations	Complexity	Post/Pre-Processing Conditions
[161]	R-AFF	Hollow cylinder—Al and Al/SiC	Rotary parts along with high torque motor Speed reduction gearbox and fixtures Abrasive: soft styrene-butadiene (SDP)/hydrocarbon processing oil 10% Particles: SiC, 220 mm, and 66.67%	0.4→0.3	Small apparatus suitable for small workpieces Very simple-shaped workpieces	Simple	Pre-processing
[5]	R-AFF	Hollow cylinder—Al and Al/SiC	Rotary parts along with high torque motor Speed reduction gearbox and fixtures Abrasive: soft styrene-butadiene (SDP)/hydrocarbon processing oil 10% Particles: SiC, 220 mm, and 66.67%	0.3→0.2	Small apparatus suitable for small workpieces Very simple-shaped workpieces	Simple	Pre-processing
[69,70]	UAAFM	Bevel gear—steel	Hydraulic system with pistons and medium cylinder Abrasive: highly viscous natural polymer (viscosity= 730 pa) with SiC particles	1.8→1.3	Applicable for steel components A narrow range of surface roughness	Complex	Pre-processing
[92]	MAF	Turned tube—Ly12 aluminum	Motor, gear, core clamper, yoke, chuck Abrasives: Al_2_O_3_/Fe (20%), TiC/Fe (20%), TiC/Fe (35%), and TiC/Fe (7%). Particles Fe, 30 # and 20%	9.6→0.2	Size-limited process Risks of damages to components	Complex	Pre-processing and post-processing
[92]	MAF	A small tube (40 mm with 30 Ø)—316L stainless steel	Motor, gear, core clamper, yoke, chuck Abrasives: Al_2_O_3_/Fe (20%), TiC/Fe (20%), TiC/Fe (35%), and TiC/Fe (7%). Particles Fe, 50 # and 35%	0.8→0.1	Size-limited process Risks of damages to components	Simple	Pre-processing and post-processing
[162]	MAF-Gel	Cylindrical road (65 mm with 15 Ø)—SKD11 and HRC60	Magnetic poles and rotary workpieces A mixture of silicone gel with steel grit (SG) and silicon carbon (SiC) Particles SiC, 2000 # and 28% and SG, 50 # and 43%	0.70→0.04	Size-limited process Risks of damages to components	Simple	Pre-processing and post-processing
[97]	AFB	Square sheets with a side dimension of 40 and 1 mm in thickness—2024 aluminium alloys	Compressor, dryer, specimen, flux meter, filters, pressure probe and gage, abrasive grain, thermometers, distributor, movement systems and computer Abrasive: alumina with SiO_2_ and Al_2_O_3_	1.0→0.4	Expensive operation Size-limited process	Complex	Post-processing
[99]	FB-AJM	Circular tube: length 200 mm and the inner diameter is 12 mm stainless steel 316L	Compressor (dryer), cyclone, fluidize bed, flowmeter, manometer and 3-way valve Abrasive: black alumina (Al_2_O_3_) with a mesh size of 24–220	1.5→0.1	Expensive operation Size-limited process	Complex	Post-processing
[101]	FB-AJM	A long and narrow tube—aluminium alloy (AA 6082 T6)	Compressor (dryer), cyclone, fluidize bed, flowmeter, manometer and 3-way valve Abrasive: angular red-brown alumina (Al_2_O_3_) with a mesh size of 24–220	4.00→0.65	Expensive operation Size-limited process	Complex	Post-processing
[163]	Drag finishing combined with fluidized bed	Rings with 10 mm long and 21 and 25 mm in inner and outer diameter—brass (Cu-30 wt.% Zn)	A rotary turntable and three satellite stations, a vertical shaft with a clamp to hold the ring, an electric motor, recycling, a fluidized bed, air supply and holders	3.0→0.2	Expensive operation Time-consuming Size-limited process	Simple and External surface	Post-processing
[142]	EMAF	Cubic shape—Al 6061	Primary coil, turning spindle, workpiece fixture and auxiliary coil Abrasive: paroline and ferriferous oxide powder with a size of 100 μm and SiC particles Electrolyte: 8.5% Na_3_PO_4_ + 3.5% Na_2_CO_3_	1.3→0.2	Size-limited process Difficult to finish complex-shaped components Risks of damages to components	Simple	Pre-processing
[146]	UEF	Cylindrical hole—AISI H13	Ultrasonic generator and tank, DC power supply, pulse-generator, pump, flowmeter and filter. Electrolyte: NaNO_3_ of 25 wt%	1.3→0.2	Size-limited process Difficult to finish complex-shaped components Risks of damages to components	Complex	Pre-processing
[164]	Electro-polishing	Rectangular shape—pure Ti foils (Grade 2, 99.5%)	A cylindrical two-electrode electropolishing cell as a cathode A mixture of methanol (99.5%, MERCK), 38ethylene glycol (MERCK), and 10 wt% perchloric acid (70%, MERCK)16 as electrolyte	0.05→0.00Mirror-like surface	Size-limited process Difficult to finish the internal surface Difficult to finish complex-shaped components Risks of damages to components	Simple	Post-processing
[103]	AFB	Axial-symmetric geometry typical of fatigue test—Ti6Al4V	A vertical fluidization column, 200 mm in height and 500 mm in diameter Inert abrasive particles Steel powder type S, mesh size 12 μm, angular steel grit, 800 HRV as abrasive	2.0→10.0	Difficult to control the flow of the abrasive particles due to the high-speed impacts	Complex	Post-processing
[68]	MAFM	SiC/B4C hybrid MMCs with aluminum as a base material	Electromagnetic coils, DC power, piston rod, 3-phase AC motor, supporting frame, hydraulic unit, magnetic field density from 0.15 T to 0.45 T; the medium was a mixture of hydraulic oil number 68, liquid silicon rubber, iron particles, and silicon carbide	1.0→2.0	Difficult to remove ferromagnetic materials	Simple	Pre-processing and post-processing
[64]	AFF	Gear—20MnCr5 alloy steel	Hydraulic cylinders containing AFF medium, hydraulic pump to produce pressure up to 20 Mpa, support frame Medium: molding clay, abrasive (SiC), and blending oil (silicon oil).	7.0→1.0	Long cycle times Expensive process Waste disposal	Simple	Pre-processing
[65]	AFM	Rectangular cube with holes—18Ni (300) maraging steel	Hydraulic pump, pressure gauge, two pistons, cooling tank, PT100 temperature sensor Abrasive particles: SiC, Al_2_O_3_, and B_2_O_3_ with a concentration of 35 wt.%.	3.0→0.5	A time-consuming process, especially for large workpieces and uneven finishing	Simple	Pre-processing and post-processing
[63]	AFF	Tube—copper	hydraulic and medium cylinders, hydraulic power pack Medium: galactomannan polymer, glycerol solution, cross-linker, and abrasive particles	6.0→1.2	Long cycle times Expensive process Waste disposal	Simple	Pre-processing
[165]	MAF	AISI H13 hot die steel	The hollow permanent magnet held, magnetic abrasive brush, workpiece fixture Medium: cast iron powder, the abrasive SiC, with a 10 W30 lubricant	0.7→0.2	Difficult to remove ferromagnetic materials	Simple	Pre-processing and post-processing
[110]	MJ-HCAF	Rectangular cube—L-PBF Inconel 625	HCAF chamber, transfer pumps, slurry tank, filtration tank, water pump Abrasive particle: sharp-edged SiC	3.0→1.0	Difficult to control the distribution of the abrasive particles	Simple	Post-processing
[144]	EMAF	Stainless steel of SUS304	Magnetic brush, DC power, flow meter, pump, magnetic pole, electrode pole Abrasive particles: iron and WA Electrolyte: NaNO_3_	0.20→0.04	Size-limited process Difficult to finish complex-shaped components Risks of damages to components	Simple	Pre-processing and post-processing
[166]	RAU-AFF	Al 6061	Ultrasonic transducer, rotary motor, wave function generator, ultrasonic horn and tip, ultrasonic booster, fluid tank The abrasive slurry was obtained from micro surface GmbH with abrasive sizes ranging from 150 to 220 μm at a concentration of 60–65%	4.8→3.4	Expensive process Limited material compatibility Limited effectiveness on deep holes and narrow channels	Complex	Pre-processing
[71]	UA-RMRAFF	Tubes—Al2024	Operation panel, 3-ɸ.A.C. motor, pump, hydraulic power unit, pressure gauge, hydraulic cylinder, limit switch, lower MRPF Cylinder, variable frequency drive (VFD), gears wheel with chain, ultrasonic power supply	0.96→0.05	Expensive process Limited material compatibility Limited effectiveness on deep holes and narrow channels	Complex	Pre-processing
[88]	MAF	Cubes and tensile bars—Inconel 718 superalloys	Magnetic grit, workpiece fixture Abrasive: diamond (120 μm)	2.70→0.15	Difficult to remove ferromagnetic materials	Simple	Post-processing
[148]	UAECDG	Hole—304 stainless steel	Pulse power supply, control cabinet, AC frequency converter, ultrasonic motorized spindle, electrolyte tank, water chiller Abrasive: diamond with number of 1200# Electrolyte: NaNO_3_	1.4→0.3	High cost Complex process Unsuitable for non-conductive materials Unsuitable for deep-hole drilling applications	Complex	Pre-processing

**Table 4:** Rating the methods based on important factors.

	Items	Roughness Improvement (20%)	Internal Surface Finishing (10%)	Size of Components (10%)	Complex-Shape Components (20%)	Possibility of Finishing the Al and Ti Products (10%)	Cost of Operation (10%)	Pre-Processing Score (20%)	Post-Processing Score (20%)	Total Pre-Processing	Total Post-Processing
Techniques											
**R-AFF**											
**U-AFF**											
**MAF**											
**MAF-Gel**											
**AFB**											
**FB-AJM**											
**Drag finishing + Fluidized bed**											
**EMAF**											
**UEF**											
**Electro polishing**											
**Score**											
**1**									**5**		

## Data Availability

The data presented in this study are openly available in the publications cited.

## Abbreviations

**Abbreviation**	**Explanation**
ABS	Acrylonitrile Butadiene Styrene
AFB	Abrasive Fluidized Bed
AFF	Abrasive Flow Finishing
AFM	Abrasive Flow Machining
AM	Additive Manufacturing
BJ	Binder Jetting
CAD	Computer-Aided Design
CAF	Cavitation Abrasive Finishing
DED	Directed Energy Deposition
DEM	Discrete Element Method
PBF	Powder Bed Fusion
L-PBF	Laser Powder Bed Fusion
EB-PBF	Electron Beam Powder Bed Fusion
ECF	Electrochemical Finishing
ECM	Electrochemical Machining
ECMP	Electrochemical Mechanical Polishing
ECP	Electrochemical Polishing
EMAF	Electrochemical Magnetic Abrasive Finishing
FBAJM	Fluidized Bed-assisted Abrasive Jet Machining
FBM	Fluidized Bed Machining
HCAF	Hydrodynamic Cavitation Abrasive Finishing
MAAFM	Magnetic Assisted Abrasive Flow Machining
MAAJM	Magnetic Assisted Abrasive Jet Machining
MAF	Magnetic Abrasive Finishing
MAP	Magnetic Abrasive Particles
MDIF	Magnetically Driven Internal Finishing
MJHCAF	Multi Jet Hydrodynamic Cavitation Abrasive Finishing
MJP	Multi-Jet Polishing
MRAFF	Magneto-Rheological Abrasive Flow Finishing
MRP	Magnetorheological Polishing
MRR	Material Removal Rate
PLA	Polylactic Acid
RAFF	Rotational Abrasive Flow Finishing
UAAFM	Ultrasonic Assisted Abrasive Flow Machining
UAECDG	Ultrasonic-Assisted Electrochemical Drill-Grinding
UAECDG	Ultrasonic-Assisted Electrochemical Drill-Grinding
UA-RMRAFF	Ultrasonic Assisted-Rotational Magnetorheological Abrasive Flow Finishing
UCAF	Ultrasonic Cavitation Abrasive Finishing
UFP	Ultrasonic Flow Polishing
